# Objective evaluation of clinical outcomes of laparoscopy-assisted pylorus-preserving gastrectomy for middle-third early gastric cancer

**DOI:** 10.1186/s12885-019-5695-0

**Published:** 2019-05-22

**Authors:** Xiang Xia, Jia Xu, Chunchao Zhu, Hui Cao, Fengrong Yu, Gang Zhao

**Affiliations:** Department of Gastrointestinal Surgery, Ren Ji Hospital, School of Medicine, Shanghai Jiao Tong University, No. 1630, Dongfang Road, Shanghai, 200127 People’s Republic of China

**Keywords:** Laparoscopic-assisted pylorus-preserving gastrectomy, pT1N0M0 gastric cancer, Gallstone, Gastric emptying, Nutritional status

## Abstract

**Background:**

Laparoscopic-assisted pylorus-preserving gastrectomy (LAPPG) is a minimally invasive function-preserving surgery for early gastric cancer. This study was designed to investigate the clinical outcomes between LAPPG and laparoscopy-assisted distal gastrectomy (LADG) by objective evaluation.

**Methods:**

A total 167 pT1N0M0 gastric cancer patients underwent LAPPG(*n* = 70) and LADG(*n* = 97) were retrospectively analyzed. By evaluating the functional advantages, objective short-term and one year follow-up outcomes were compared.

**Results:**

There is no significant difference in perioperative clinical characteristics as well as pathologic results between LAPPG and LADG group while the cost is higher in latter(*p* = 0.004). The Clavien–Dindo grade II or higher complications were 15.7 and 13.4% in LAPPG and LADG group respectively(*p* = 0.824). In one year follow-up, nutritional status was significantly better in LAPPG group accompanied by better pylorus function preserving.

**Conclusion:**

LAPPG is an acceptable surgical procedure for pT1N0M0 middle portion gastric cancer patients in terms of nutritional and economic advantage.

**Trial registration:**

Chinese Clinical Trial Registry (ChiCTR-PIC-17012358, Date of Registration:2017-08-14).

## Background

Gastric cancer is the third most frequent cause of cancer related death and the fifth most common cancer worldwide with nearly 951,000 newly diagnosed patients as well as 723,000 death in 2012 [1]. In recent years, due to the popularization of heath screening programs and development of high-quality endoscopic instruments, the number of early gastric cancer (EGC) has been gradually increasing. Furthermore, because EGC usually owns a low metastatic incidence and favorable survival rates, surgeons have started to place special emphasis on function-preserving and nutritional status improvement for those patients [2, 3].

Compared to traditional open gastrectomy, laparoscopic gastrectomy has the superiority of the minimally invasive approach, such as less postoperative pain, better cosmetic results, early recovery of bowel function and a rapid recovery to regular activity [4–6]. Moreover, laparoscopy-assisted distal gastrectomy (LADG) has been a standard surgical procedure for Stage I gastric cancer [7]. Nevertheless, because the extent of distal gastrectomy is identical in laparoscopic and open surgery, the long-term outcomes and postgastrectomy symptoms of LADG, including dumping syndromes or remnant gastritis were similar to open distal gastrectomy.

Pylorus-preserving gastrectomy (PPG) is a typical operation of function-preserving for EGC located in the middle portion of the stomach [3, 8]. This surgery was first introduced to treat benign gastric ulcers in 1967 by Maki et al. [9]. And in the current version of the Japanese Gastric Cancer Treatment Guidelines, PPG is described as a modified procedure for cT1N0M0 EGC in the middle portion of the stomach [10]. Based on the advantages of laparoscopy and PPG, LAPPG started to apply in some EGC patients by some gastrointestinal surgeons. But to date, no randomized controlled trial was reported to compare the perioperative outcomes and long-term nutritional status between LAPPG and LADG.

In this study, we present the short-term outcomes and one-year follow up postoperative nutritional status including gallstone formation and gastric emptying evaluation of LAPPG and LADG.

## Methods

### Patients

Between April 2015 and December 2017, A review of medical records including clinical and pathologic reports at Department of Gastrointestinal Surgery, Ren Ji hospital, School of Medicine, Shanghai Jiao Tong University identified 167 pT1N0M0 patients underwent LAPPG and LADG. The indication for LAPPG was cT1N0M0 gastric cancer located in the middle or lower-third of the stomach without evidence of regional lymph node metastasis, more than 5 cm proximal to the pyloric ring and with a maximum diameter less than 5 cm, while other cT1N0M0 gastric cancer located in the middle or lower-third of the stomach without evidence of regional lymph node metastasis were included in LADG group. All patients received an upper gastrointestinal endoscopy, pathological biopsy, computed tomography, and sometimes endoscopic or abdominal ultrasonography. cT1N0M0 gastric cancer patients would be excluded if they were found later to be pN1. Whether a patient was treated by LAPPG or LADG was decided by patient request when tumor located more than 5 cm proximal to the pyloric ring and with a maximum diameter less than 5 cm, and otherwise by attending surgeon preference. Patients who were candidates for endoscopic resection were not included in the study. No patients were received chemotherapy in this study. It was likewise registered in the Chinese Clinical Trial Register (ChiCTR), a primary register of the WHO International Clinical Trials Registry Platform (SN. ChiCTR-PIC-17012358).

### Surgical procedures

After general anesthesia, patients were laid in the supine, reverse Trendelenburg position with leg elevation. A 12-mm trocar for the camera port was inserted through the umbilical port and a pneumoperitoneum was created by CO_2_ inflation at the pressure of 12 mmHg. Under the view of the laparoscopic image, a 12 mm trocar and a 5 mm trocar were inserted in the left upper and lower quadrants while two 5 mm trocar were inserted in the right upper and lower quadrants respectively. Then falciform ligation was lifted by prolene stay sutures.

### LAPPG and LADG

LAPPG partially preserved the greater omentum and cut the omentum 3-4 cm inferior to the gastroepiploic arcade. Kocher’s maneuver to mobilize the duodenum was done to minimize the tension for the gastro-gastric anastomosis. Lymph node included No. 4d(right astroepiploic artery) and No. 4sb(left gastroepiploic artery) were dissected in this procedure. Then the origin of the right gastroepiploic artery and vein were divided carefully and the ligation of the right epiploic vessels should be distal to the branches of infrapyloric vessels to maintain the blood supply to pyloric cuff followed by No. 6 lymph nodes dissection. The No. 5 lymph nodes were left intact with the right gastric vessels was ligated approximately 3 cm apart from the pylorus. Subsequently No. 7, 8a, 9 and partial 11p lymph nodes were dissected as well as ligation of the left gastric artery. The No. 1, 3 lymph nodes were removed followed by preservation of the hepatic branch of the vagus. After lymphadenectomy, the stomach was extracted through a 5 cm midline incision. The distal part of the stomach was resected leaving at least 3 cm antral cuff. The proximal portion of the stomach was kept with a 3 cm proximal margin for an oncologically safe margin. A two-layer extracorporeal handsewn gastrogastroanastomosis was then performed.

LADG procedure has been described previously [11]. In brief, trocar placement and laparoscopic procedures were similar to LAPPG, except for the dissection of suprapyloric and infrapyloric lymph nodes and preserving the hepatic branch of the vagus nerve. Suprapyloric lymph nodes were completely dissected with the division of the root of the right gastric vessels. Infrapyloric lymph nodes were completely dissected with the division of the root of the right gastroepiploic and infrapyloric vessels. The celiac branch of the vagus nerve was dissected during the dissection of the lymph nodes along the celiac artery. The posterior wall of the remnant stomach and the duodenal stump was mechanically anastomosed with Bilroth I anastomosis.

### Postoperative data and surveillance

Demographic and clinicopathologic data were reviewed from our medical records. The microscopic classification of tumors was based on the 3rd English edition of the Japanese Classification of Gastric Carcinoma [12] and the 7th edition of the International Union Against Cancer/American Joint Committee on Cancer TNM staging system [13]. Postoperative complications were categorized according to the Clavien–Dindo classification [14].

In follow up protocol, all patients were examined by physical and blood cell counts, blood chemistry tests including hemoglobin, serum total protein and serum albumin every 6 months. Meanwhile, BMI calculation, computed tomography, abdominal ultrasonography for gallstone, gastric emptying test, tumor markers and gastroscopy were performed at every 6 months hospital visits.

### Gallbladder and gastric emptying examination

Gallbladder volumes were calculated using the ellipsoid method using the formula V = 0.52 (L × W × H), where W is the gallbladder width, H is height and L is axial length [15]. After an overnight fasting, the gallbladder basal volume was measured in the morning with patients in the supine position turned partially on their right side. The residual volume was measured one hour later after eating two fried eggs. The gallbladder emptying rate was considered the difference between basal volume and residual volume/ basal volume× 100.

After 12 h fasting, patients underwent gastric emptying scintigraphy technique by eating a meal of 40 g of black sesame paste and a 17.5 g cheese mixed with 300 ml water. The meal was labeled with ^99m^Tc-DTPA 2 mCi. Gastric emptying was followed by continuous imaging at 0, 5, 10, 15, 20, 25, 30, 60 and 90 min per frame. We set a linear rate of emptying to the data from 0 to 90 min and obtained an extrapolated half-time of emptying (normal t½ =65–85 min). Normal values for this meal and imaging methodology were obtained from 20 normal volunteers (age range: 26–60) in 2014 at our hospital (not published data).

Because different feeding times and food components would affect these two examinations, the gastric and gall bladder emptying tests were performed in two separate visits.

### Statistical analysis

SPSS version 13.0 software (SPSS, Chicago, IL, USA) for windows was utilized to perform statistical analyses. Baseline characteristics were compared by Mann–Whitney U-test for continuous variables, and the chi-square test was used for categorical variables. *P* values less than 0.05 were considered significant.

## Results

### Patients’ clinicalpathological characteristics and perioperative outcomes

Detailed information concerning those 167 patients was presented in Tables 1 and 2. The mean age, gender, mean BMI, preoperative comorbidity, ASA (American Society of Anesthesiologists) and ESD history were similar between LAPPG and LADG groups (Table 1). The perioperative outcomes are summarized in Table 2. The mean operation time and blood loss were comparable between two groups. For oncologic safety, there is no significant difference about the mean maximum tumor diameter, proximal margin, distal margin and no. of examined lymph between LAPPG and LADG group. The postoperative pathological examination revealed that all cases fulfilled pT1N0M0 criterion. Moreover, no difference was observed in median postoperative hospitalization, postoperative first flatus, postoperative gastric tube decompression, postoperative fluid diet start and Clavien Dindo Grade II or high complication. Nevertheless, hospitalization expenses were significantly less in LAPPG compared to LADG(*P* = 0.004).

**Table 1:** Characteristics of patients undergoing LAPPG and LADG

Variables	LAPPG(*n* = 70)	LADG(*n* = 97)	*P* value
Mean age (years)^a^	56.8 ± 10.9	57.5 ± 12.1	0.667
Gender			1.000
Male	46 (65.7%)	63 (64.9%)	
Female	24 (34.3%)	34 (35.1%)	
Mean BMI (Kg/m^2^)^a^	22.3 ± 2.3	22.7 ± 4.8	0.495
Preoperative comorbidity			0.800
None	61 (87.1%)	87 (89.7%)	
Hypertension	6 (8.6%)	7 (7.2%)	
Diabetes	3 (4.3%)	3 (3.1%)	
ASA			0.794
I-II	64 (91.3%)	87 (89.7%)	
III	6 (8.7%)	10 (10.3%)	
ESD preoperatively			0.523
Yes	3 (4.3%)	7 (7.2%)	
No	67 (95.7%)	90 (92.8%)	

*BMI* body mass index, *ASA* American Society of Anesthesiologists, *ESD* endoscopic submucosal dissection

^a^Values are shown as mean ± standard deviation

**Table 2:** Perioperative outcomes and pathologic results about LAPPG and LADG

Variables	LAPPG(*n* = 70)	LADG(*n* = 97)	*P* value
Mean operation time (min)^b^	220.5 ± 17.2	223.8 ± 28.1	0.216
Mean blood loss (ml)^b^	46.9 ± 49.6	48.5 ± 51.1	0.830
Mean maximum tumor diameter (cm)^b^	1.8 ± 0.7	1.8 ± 0.7	0.934
Mean proximal margin (cm)^b^	2.9 ± 0.8	3.0 ± 0.8	0.233
Positive proximal margin rate	0 (0%)	0 (0%)	1.000
Mean distal margin (cm)^b^	3.8 ± 1.4	3.6 ± 1.7	0.265
Positive distal margin rate	0 (0%)	0 (0%)	1.000
Mean total no. of examined lymph nodes^b^	22.4 ± 5.3	23.2 ± 5.5	0.337
Median postoperative hospitalization (days, range)	8 (7–30)	8 (7–27)	0.199
Median postoperative first flatus (days, range)	4 (3–5)	4 (2–5)	0.571
Median postoperative gastric tube decompression (days, range)	4 (2–20)	3 (2–19)	0.656
Median postoperative fluid diet start (days, range)	5 (4–21)	5 (4–19)	0.346
Postoperative complication^a^ ≥ II (no. of patients)	11 (15.7%)	13 (13.4%)	0.824
Gastric stasis	4 (5.7%)	2 (2.1%)	
Anastomotic leak	1 (1.4%)	3 (3.1%)	
Abdominal absces	1 (1.4%)	2 (2.1%)	
Respiratory complication	5 (7.1%)	6 (6.2%)	
Differentiation			0.215
Well or moderate differentiation	47 (67.1%)	68 (76.4%)	
Poorly differentiation or Signet ring cell	23 (32.9%)	21 (23.6%)	
pT category			1.000
T1a	33 (47.1%)	45 (46.4%)	
T1b	37 (52.9%)	52 (53.6%)	
Hospitalization expenses (Ten thousands)^b^	4.6 ± 0.5	5.3 ± 0.4	0.004

^a^Acording to the Clavien–Dindo classification

^b^Values are shown as mean ± standard deviation

### Postoperative nutrition, gallbladder contraction and gastric emptying

The median follow up period was 24 months. As shown in Table 3, the levels of serum total protein, albumin, hemoglobin and BMI were significantly improved after LAPPG at 12 months compared to that of LADG. Table 4 summarized the functions of gallbladder contraction and gastric emptying in all patients. Gallstones developed in 5 LAPPG patients (7.1%) and 5 LADG patients (5.2%) without significant difference(*p* = 0.744). However, the mean gallbladder emptying rate was significantly higher in LAPPG group (34.04 ± 15.3 vs 27.32 ± 15.9) while the mean time to half gastric emptying (110.11 ± 44.5 vs 92.51 ± 54.6) and percentage of retention in the stomach at 120 min(46.27 ± 20.5 vs 40.27 ± 21.9) were significantly more than those of LADG group(*p*<0.05).

**Table 3:** Nutritional status and BMI between LAPPG and LADG in one year follow-up

(Preoperative—postoperative)/preoperative	LAPPG**(***n* = 70**)**	LADG(*n* = 97)	*P* value
Serum total protein level(%)	11.30 ± 18.0	5.9 ± 13.0	0.046
Serum albumin level (%)	17.20 ± 25.6	10.06 ± 15.4	0.048
Hemoglobin(%)	−1.09 ± 4.0	−2.73 ± 3.8	0.014
BMI(%)	−2.53 ± 3.1	−3.64 ± 3.4	0.048

Values are shown as mean ± SD, *SD* standard deviation, *BMI* body mass index

**Table 4:** Gallstone formation, gallbladder emptying and gastric emptying between LAPPG and LADG in one year follow-up

Variables	LAPPG(*n* = 70)	LADG(*n* = 97)	*P* value
Gallstone(n)	5 (7.1%)	8 (8.2%)	1.000
Gallbladder emptying rates(%)	34.04 ± 15.3	27.32 ± 15.9	0.012
Time to half gastric emptying (min)	110.11 ± 44.5	92.51 ± 54.6	0.032
Retention at 120 min of stomach(%)	46.27 ± 20.5	40.27 ± 21.9	0.042

Values are shown as mean ± SD, *SD* standard deviation

## Discussion

PPG has been proved to be a safe operation for early gastric cancer patients with excellent short and long-term prognosis [16, 17]. LAPPG, a less invasive operation compared to PPG, not only had several advantages in early postoperative outcomes, such as reducing intraoperative blood loss, postoperative pain, hospital stay and accelerate bowel function recovery and fluid oral intake [18], but also could ameliorate early dumping syndromes, body weight loss and duodenogastric reflux although those patients might more frequently experience delayed gastric emptying, abdominal fullness and gastro-esophageal reflux disorder than LADG in short term [8, 19, 20].

Since 2011, our surgical team started to apply laparoscopic approach to treat early gastric cancer, including LADG with D2 lymphadenectomy. After accumulating enough clinical experience, we started to apply LAPPG for cT1N0M0 gastric cancer patients whose tumor located in the middle portion of the stomach. In this study, we would like to share some experiences regarding comparisons of short-term clinical outcomes as well as one year follow up surveillance of LAPPG and LADG in our institution. Furthermore, this is the first evaluation worldwide using objective methods such as gallbladder contraction and gastric emptying.

As shown in Table 1, the clinical characteristics of patients in LAPPG and LADG were similar. As far as perioperative outcomes and pathologic results are concerned, the mean operation time for LAPPG was 220.5 ± 17.2 min and introperative blood loss was 46.9 ± 49.6 ml, which were similar to other studies [18, 21, 22] and were not significantly different from those of LADG. Our results also showed that the postoperative recovery such as median hospitalization, postoperative fluid diet start, postoperative complications rate were not significantly different between LAPPG and LADG groups. Gastric stasis or delay emptying is a typical complication and might be the greatest pitfalls of PPG. In our study, 4 patients (5.7%) in LAPPG group experienced gastric stasis while the number of LADG group is 2(2.1%) but there is no significant difference(*p* = 0.239). Also, our gastric stasis rate of LAPPG was comparable to the reports that ranged from 5.2 to 10.3% [8, 21]. The reasons for gastric stasis remain unclear. The possible mechanism was pylorus edema or nerve dysfunction induced by mechanical and chemical injury such as thermal insult from ultrasonic energy device [18, 23]. The four gastric stasis patients in our study were all cured by conservative management such as gastric tube decompression, parenteral alimentation and fasting. Bae et al. [24] suggested that the standardization of the surgical procedure such as preserving blood flow and the hepatic branch of the vagus nerve could reduce the severity of gastric stasis. Zhu et al. [23] reported introperative manual dilation of pylorus help to prevent pyloric stenosis. However, those above skills were not tested in a randomized trial and thus a randomized controlled trial was needed to determine these outcomes.

In order to minimize interviewers’ bias and obtain steady reproducibility, objective data including laboratory findings and BMI, instead of questionnaires from patients were collected in the outpatient clinic one year follow-up. As a result, the values of nutritional status such as serum total protein, albumin, hemoglobin and BMI in LAPPG group were elevated with significant difference as compared to those of LADG group. We speculated that the larger size functional gastric reservoir, preservation of pyloric cuff as well as hepatic and pyloric branches of vagus nerve, the retained gastric acid secretion and a longer gastric emptying for ferric iron and nutrition absorption after food mixed with gastric acid acted the beneficial factors in improving nutritional status and maintain BMI [8, 21, 25, 26].

**Fig. 1:**
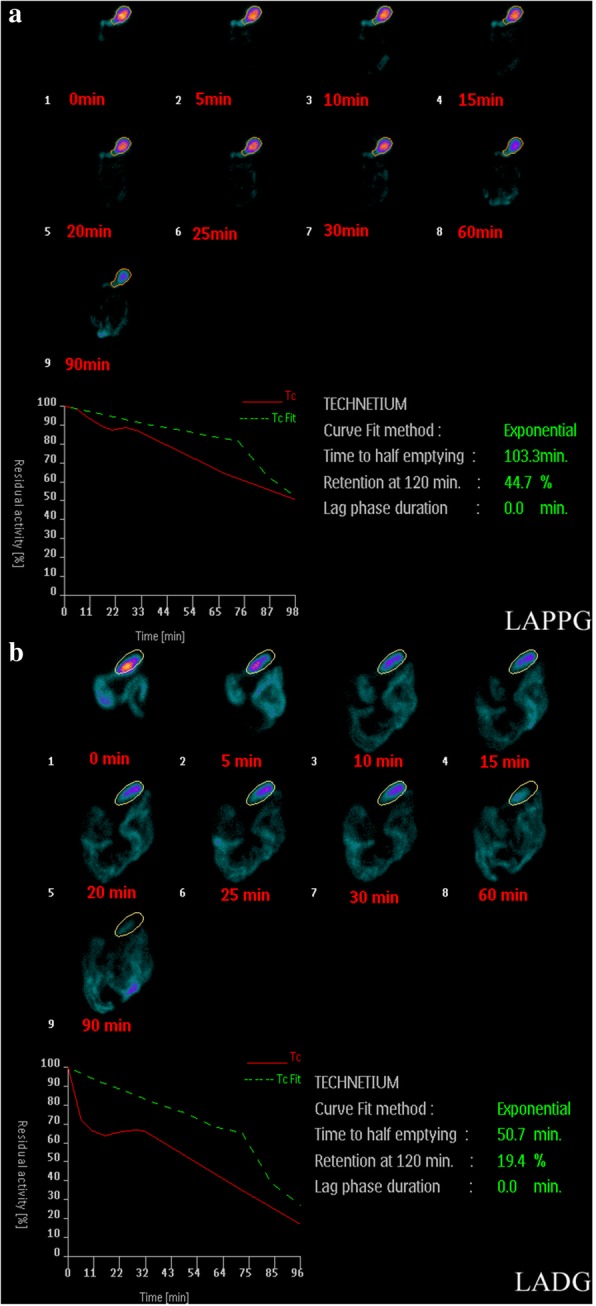
Typical gastric emptying examination for LAPPG (**a**) and LADG (**b**) patients at one year follow-up surveillance respectively. **a** the time to half gastric emptying is 103.3 min and the retention at 120 min of stomach is 44.7%. **b** the time to half gastric emptying is 50.7 min and the retention at 120 min of stomach is 19.4%

The gastric emptying examination in one year follow-up further demonstrated the significantly longer time to half gastric emptying and more food retention at 120 min in LAPPG group than those in LADG group (Table 4 and Fig. 1).

Gallstone is one of common complications after gastrectomy [27]. The incidence of gallstone in LAPPG group was 7.1% and the gallbladder emptying rate was 34.04 ± 15.3% in one year follow-up, both of which outdoes LADG group (8.2% and 27.32 ± 15.9%, respectively). The pathophysiology of gallstone information after gastrectomy was regarded as vagal nerve resection, nonphysiological reconstruction of the gastrointestinal tract and decreased secretion of cholecystokinin [28, 29]. LAPPG could preserve hepatic and pyloric branches of the vagus nerve to keep pyloroduodenal myoneural continuity and maintain Oddi sphincter contraction and gallbladder emptying [30]. Findings from Imada et al. [25] and ours both proved that gallbladder functions in pylorus-preserving gastrectomy patients were much better than distal gastrectomy patients.

The most important limitation of our study was associated with its non-randomized, retrospective design. Nonetheless, this was the first report for comparing LAPPG to LADG in Chinese early gastric cancer patients and based on these retrospective experiences and data, our prospective randomized controlled trials (ClinicalTrials.gov Identifier: NCT02936193) are approved and starting to recruiting eligible patients which will present more detailed and persuasive studies. Secondly, the period of follow up was a little short considering the usual long-term survival analysis. However, the primary endpoint of our study was to evaluate clinical outcomes of surgery and nutrition objectively other than oncological safety which had already been tested and proved [3]. Finally, our study did not use questionnaires to evaluate QOL such as PGSAS-45 which was established by the Japanese Postgastrectomy Syndrome Working Party to measure postgastrectomy syndromes. That is because most of these subjective feelings from our small sample size study showed large dispersion degree.

## Conclusion

In conclusion, our study demonstrated objective clinical outcomes of LAPPG for pT1N0M0 gastric in Chinese patients. By strict inclusion criteria, LAPPG could be a recommended surgical procedure for early gastric cancer located in the middle portion of the stomach. Ongoing clinical trials in our institution (ClinicalTrials.gov Identifier: NCT02936193) and Korean (ClinicalTrials.gov Identifier:NCT No.02595086) are expected to claim the indications and superiority of LAPPG.

## Abbreviations

ASA: American Society of Anesthesiologists
ChiCTR: Chinese Clinical Trial Register
LADG: Laparoscopy-assisted distal gastrectomy
LAPPG: Laparoscopic-assisted pylorus-preserving gastrectomy
PPG: Early gastric cancer (EGC); Pylorus-preserving gastrectomy
